# Gonadal bacterial community composition is associated with sex-specific differences in swamp eels *(Monopterus albus)*

**DOI:** 10.3389/fimmu.2022.938326

**Published:** 2022-08-24

**Authors:** Kaifeng Meng, Xing Lin, Hairong Liu, Huijie Chen, Fei Liu, Zhen Xu, Yonghua Sun, Daji Luo

**Affiliations:** ^1^ State Key Laboratory of Freshwater Ecology and Biotechnology, Institute of Hydrobiology, The Innovative Academy of Seed Design, Hubei Hongshan Laboratory, Chinese Academy of Sciences, Wuhan, China; ^2^ College of Fisheries, Huazhong Agricultural University, Wuhan, China; ^3^ College of Advanced Agricultural Sciences, University of Chinese Academy of Sciences, Beijing, China

**Keywords:** bacterial community, sex-specific differences, gonads, swamp eel (*monopterus albus*), 16S rRNA gene sequences

## Abstract

Organisms are colonized by microorganism communities and play a pivotal role in host function by influencing physiology and development. In mammals, bacterial community may alter gonadal maturation and drive sex-specific differences in gene expression and metabolism. However, bacterial microbiota diversity in the gonads of early vertebrates has not been fully elucidated. Here, we focused on the swamp eel (*Monopterus albus*), which naturally undergoes sex reversal, and systematically analyzed the bacterial microbiota profiles between females and males using 16S rRNA gene sequences. Specifically, the microbial abundance and community diversity of gonads in males were higher than in females. Although Proteobacteria, Firmicutes, Bacteroidetes, and Actinobacteria were characterized as the dominating phyla in ovary and testis, the relative abundance of Firmicutes was significantly higher in males than females. Detailed analysis of the microbial community revealed that *Bacilli* were the dominant bacteria in ovaries and *Clostridium* in testes of *M. albus*. More importantly, we proposed that differences in the microbial composition and distribution between ovaries and testes may be linked to functional categories in *M. albus*, especially metabolism. These findings represent a unique resource of bacterial community in gonads to facilitate future research about the mechanism of how microbiota influence sex-specific differences and sex reversal in vertebrates.

## Highlights

1. Microbial abundance and community diversity of gonads in *M. albus* are present at higher levels in males than females;
2. *Bacilli* may be the dominant bacteria in ovaries and *Clostridium* in testes of *M. albus*;
3. Bacterial community may be linked to gonadal development and function in *M. albus*.

## Introduction

Vertebrate surfaces are inhabited by dense and complex microbial populations characterized by remarkable dynamism and exceptional stability (1, 2). Shaped by millennia of evolution, beneficial and balanced relationships have developed between hosts and microbes, where microbes play essential roles in many biological functions, including development, nutrition, and immune responses (3–5). Thus, a balanced microbiome helps maintain normal host physiology, and imbalances may be linked to physiological disorders (6). Evidence is accumulating about the microbiome’s roles in various sexual dimorphisms and sex-specific rhythms in mammals (7). The commensal microbial community alters testosterone, a gonadal steroid related to the gonadal transition in species that undergo sex reversal (8, 9). Besides that, it is bacterial microbiota (*Lactobacillus* and *Clostridia*) that is responsible for semen quality and fertility status in mammals (10, 11). Changes in the abundance of multiple bacteria from the Bacteroidetes and Firmicutes phyla are associated with polycystic ovary syndrome (PCOS) (12). However, how sex-specific differences influence bacterial microbiota diversity in early vertebrates has not been fully elucidated.

Water is a microbial-rich environment that promotes bacterial growth compare to air. In other words, the vertebrate transition from water to land likely affected the relationships between hosts and their microbial community (13). Thus, studies on bacterial microbiome of fish may provide a broader understanding of vertebrate microbiomes due to the complexity and diversity of the microbes in fish habitats. At present, most studies have focused on the compositions of bacterial communities on teleost mucosal surfaces and found that different tissues are inhabited by unique microbial communities and proportions of specific bacteria (13, 14). In addition, it was illustrated that gender was one of the factors influencing the intestinal microbial composition in *D. rerio*, *M. albus*, *B. pectinirostris* and *C. guichenoti* (15–18). A recent study identified that the cloaca of Atlantic salmon (*Salmon salar*) was an additional teleost mucosa-associated lymphoid tissue (MALT), and we hypothesized that gonads connecting the cloaca also harbor abundant microbial communities as a matter of course (19, 20). Although preliminary descriptions of testicular microbiota compositions in zebrafish have shown that *Pseudomonas*, *Lactobacillus*, and *Bifidobacterium* are the main genera (21), the gonadal microorganisms driven by sex differences remain to be elucidated in most teleost species.

The swamp eel (*Monopterus albus*) is a typical protogynous hermaphrodite fish that undergoes sexual reversal from female to male during its lifecycle (22). The sex reversal process involves coordinated transformations across multiple factors, including primordial germ cells and neuroendocrine and molecular axes (23–27). Several studies have reported that the bacterial community is critical to sex-specific differences in gene expression and metabolism in mammals (7). These findings suggest a new mechanism that the bacterial community might affect host sexual maturation (28). Sexual fate can no longer be considered an irreversible deterministic process in many fish. Exploring whether the bacterial community is required for the underlying biological processes of sex is a worthy scientific endeavor. In brief, comparing the microbial differences in *M. albus* from the perspective of the essential tissues, gonads, will increase our understanding of sex-specific differences.

In the present study, *M. albus* of the same age were chosen as our experimental animal and kept under identical conditions (e.g., environment, diet). Sex identification showed that male individuals appeared in the population of *M. albus* through artificial propagation. Moreover, dramatic differences of the bacterial composition and distribution between testis and ovary in *M. albus*, suggesting that gonadal microbiota may be sex-specific differences. In addition, key bacterial community in ovary and testis were screened, providing favorable practical significance for the production of *M. albus*.

## Materials and methods

### Fish maintenance

**Figure 1:**
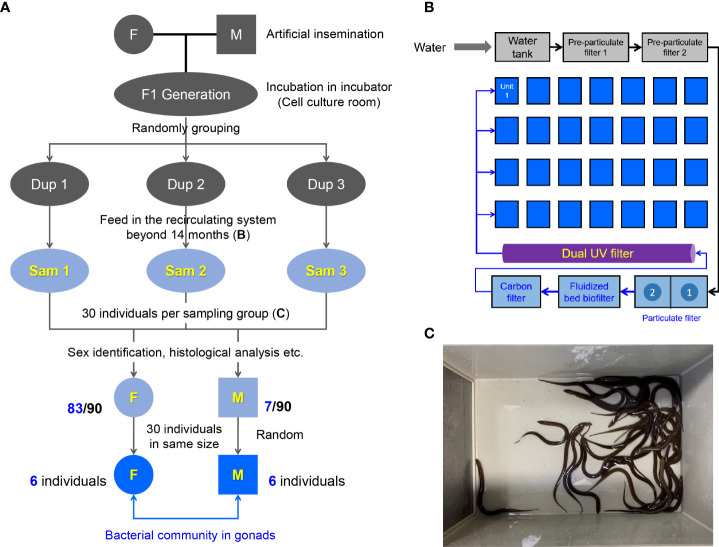
Design and sampling of this study. **(A)** Generation of sibling population in *M. albus* and experimental sampling design. **(B)** Schematic diagram of the recirculating system. **(C)** 30 individuals were randomly selected as the sampling group from the unit of the recirculating system.

The *M. albus* was originally purchased from Baishazhou Agricultural Market, Wuhan, China (22). After domestication under laboratory conditions, healthy and mature *M. albus* were selected as parents to generate the F1 sibling generation offspring using artificial insemination (**Figure 1A**). After artificial insemination, embryos were incubated in the incubator of cell culture room until the mouth-opening stage of larval *M. albus*. Hatching occurred 7 dpf (days post fertilization) at 27 ± 1°C. After the juveniles could eat the bloodworms, they were randomly divided into 3 groups (Dup1, Dup2 and Dup3 as parallel experiments) and transferred to the indoor circulating system involving appropriate temperature (25-28°C), pH value (7.2-8.0), and dissolved oxygen over 14 months (**Figure 1B**). Frozen bloodworm at a rate of 0.5%-1% body weight was fed twice a day (9:00 a.m. and 4:00 p.m.) during the whole feeding process in the indoor recirculating system. Experimental fish (30 individuals per group) were randomly selected from each Dup group and transferred to plastic boxes one week prior to sampling (Sam1, Sam2 and Sam3 group as experimental duplication) (**Figure 1C**). During this period, fish were terminated from feeding and half of the culture water was replaced with ultrapure water every three days. After sex identification, 30 individuals of similar size including females and males were screened according to the size of fish for the subsequent study. All animal procedures were carried out in accordance with the Guiding Principles for the Care and Use of Laboratory Animals and approved by the Institute of Hydrobiology, Chinese Academy of Sciences.

### Sample collection

Before sampling, the water of fish living (3 replicate water samples) was firstly collected for 16S rRNA gene sequencing to obtain the background composition of bacterial community in water environment **(** **Supplementary Figure 1** **)**. Subsequently, the *M. albus* were anesthetized with MS-222 and the length and weight were measured. After the fish body surface was swabbed with 75% ethanol, the blood then was collected and immediately transferred to a 2 ml sterile blood collection vessel and centrifuged at 3000 rpm for 30 min to obtain serum. After sterile dissection, the gonads were collected, weighed, and divided into three parts. One of them was fixed immediately at 4% (v/v) neutral buffer paraformaldehyde for sex identification. The other part was collected in sterile micro-centrifuge tubes with 1 ml TRIZol for RNA extraction and quantitative real-time PCR (qRT-PCR). The last part was stored in sterile freezing tubes for bacteria 16S rRNA gene sequencing. The weight relationship between gonads and body was represented by GSI (Wgonad/Wbody×100%). All of these tissues collected for RNA or DNA analyses were immediately frozen with liquid nitrogen and stored at -80°C for further study.

### Measurement and analysis of sex hormones

The serum samples were thawed on ice, and separated into two centrifuge tubes. One of the duplicate samples was used for estradiol analysis and the other for testosterone. The concentrations of estradiol and testosterone in serum were determined by using fish-specific enzyme-linked immunosorbent assay (ELISA) kits (MEIMIAN, China) according to the manufacturer’s instructions. Briefly, the serum was incubated in microelisa stripplate at 37°C for 30 min. After washing five times with wash solution, 50 μl HRP-Conjunction reagent was added and incubated at 37°C for 30 min. The 50 μl chromogen solution was added for 10 min followed by 50 μl stop solution. The OD values of each well were detected at 450 nm absorbance (MD SpectraMax M5 Microplate Reader, USA) after adding the stop solution.

### Histology and light microscopy studies

After fixed in 4% neutral formalin buffer more than 24 hours, the gonads were dehydrated through a series of ethanol gradient, washed with xylene, embedded in paraffin, and then sectioned into 4 μm pieces. The paraffin pieces were stained with classic hematoxylin and eosin (H&E) as follows: dewaxed in xylene, rehydrated through a graded ethanol series, stained the nucleus with hematoxylin solution and cytoplasm with eosin solution, finally sealed by resin. Subsequently, images were acquired in a microscope (Nikon, Japan) using the CaseViewer software.

### Micro-CT scanning

To distinguish the anatomical relationships between the gonads and the gut in *M. albus*, we performed the CT investigations. Specifically, *M. albus* was fixed in 4% neutral formalin buffer more than 24 hours, then dehydrated through a series of ethanol gradient for 4 days. After gradient dehydration, *M. albus* was immersed in phosphotungstic acid (6 mg/ml) diluted with ethanol at 37°C for 10 days. Subsequently, *M. albus* was subjected to fixed bed and scanned with Skyscan High Resolution Micro-CT Imaging System (Bruker, Belgium). The Data Viewer software and CTvox software was used for post-processing and capture of pictures **(** **Supplementary Figure 2** **)**.

### RNA extraction and quantitative real-time PCR analysis

Total RNA was extracted from gonads which were homogenized in 1 mL TRIZol (Invitrogen, USA) by shaking (60 HZ for 1 min) with 3 mm beads following the manufacturer’s instructions. To normalize gene expression levels for each sample, equivalent amounts of the total RNA (1000 ng) were used for cDNA synthesis with the SuperScript first-strand synthesis system for qRT-PCR (Monad, China) in a 20 μl reaction volume. The synthesized cDNA was diluted 5 times and then was used as a template for qRT-PCR analysis. The qRT-PCR was performed in a Bio-Rad CFX96 Touch Detection System (BioRad, USA) by using the 2× SYBR qPCR Master mix (Monad, China) as the following conditions: 95°C for 5 min, followed by 40 cycles at 95°C for 10 s and at 58°C for 30 s. Relative fold changes of genes were calculated by the methods of -ΔΔCt and the housekeeping gene elongation factor (EF-1α) was used as control gene for normalization of expression. Primers used for qRT-PCR are listed in **Supplementary Table S1**.

### DNA extraction and PCR amplification

Following the manufacturer’s instructions, the total genomic DNA from twelve gonads (six testis and six ovaries) was extracted using MagPure Stool DNA KF kit B (Magen, China). Then the quantity and quality of extracted DNA were measured with a Qubit Fluorometer by using the Qubit dsDNA BR Assay kit (Invitrogen, USA) and checked by running aliquot on 1% agarose gel, respectively.

Variable regions V3–V4 of bacterial 16S rRNA gene was amplified with universal PCR primers, 338F (5’-ACTCCTACGGGAGGCAGCAG-3’) and 806R (5’-GGACTACHVGGGTWTCTAAT-3’). Both forward and reverse primers were tagged with Illumina adapter, pad, and linker sequences. PCR enrichment was performed in a 50 μl reaction containing 30ng template, fusion PCR primer, and PCR master mix as following conditions: 94°C for 3 min, followed by 30 cycles of 94°C for 30 s, 50°C for 45 s, 72°C for 45 s and final extension for 10 min at 72°C. The PCR products were purified with AmpureXP beads and eluted in Elution buffer. Libraries were qualified by the Agilent 2100 bioanalyzer (Agilent, USA). The validated libraries were used for sequencing on the Illumina HiSeq 2500 platform (BGI, China) following the standard pipelines of Illumina, and generating 2 × 300 bp pairedend reads.

### Illumina MiSeq sequencing and bioinformatics analysis

Raw reads were filtered to remove adaptors and low-quality and ambiguous bases, and then paired-end reads were added to tags by the Fast Length Adjustment of Short reads program (FLASH) to get the tags. The tags were clustered into OTUs with a cutoff value of 97% using UPARSE software and chimera sequences were compared with the Gold database using UCHIME to detect. Then, OTU representative sequences were taxonomically classified using Ribosomal Database Project (RDP) Classifier with a minimum confidence threshold of 0.6, and trained on the RDP Release16 database by QIIME (29). The USEARCH global was used to compare all tags back to OTU to get the OTU abundance statistics table of each sample. Alpha and beta diversity were estimated by MOTHUR and QIIME at the OTU level, respectively. Principal component analysis (PCA) in OTUs was plotted with R package “ade4”. Principal Coordinate Analysis (PCoA) was performed by QIIME (v1.8.0). Nonmetric multidimensional scaling ordination (NMDS) was performed by R package. UPGMA cluster was performed by phytools and R package version 3.5.1. Wilcox Test results of species difference were performed by R/Bioconductor package DESeq. Significant differences were considered between the two groups when p-vaule and FDR values were less than 0.05. LEfSe cluster or LDA analysis was conducted by LEfSe. KEGG functions were predicted using the PICRUSt software (30). Barplot and heatmap of different classification levels were plotted with R package v3.4.1 and R package “gplots”, respectively. The sequence information for this study has been uploaded to NCBI with the accession number PRJNA832434.

### Identification and analysis of gonadal bacteria

To verify the key bacteria in the gonadal tissue, we sampled the gonadal tissue of *M. albus* under the same background. After homogenization by bead beating for 2 min at 60 Hz, 100 μl homogenate was absorbed onto the plate preparation of Brain Heart Infusion Agar. Subsequently, single colonies were selected to culture for further enrichment post nearly 12 hours. Bacterial universal primers were used for further amplification in a 50 μl reaction including 25 μl 2× PCR master mix, 2 μl bacteria template, and 2 μl primers as following conditions: 94°C for 10 min, followed by 35 cycles of 94°C for 30 s, 58°C for 30 s, 72°C for 1 min and final extension for 10 min at 72°C. After being analyzed by agarose gel electrophoresis and photographed, PCR products were purified and sequenced.

### Statistical analysis

An unpaired Student’s *t*-test (Prism version 8.0; GraphPad) was used for analysis of differences between groups. *P* values of 0.05 or less were considered statistically significant.

## Results

### Sex identification of *M. albus*

**Figure 2:**
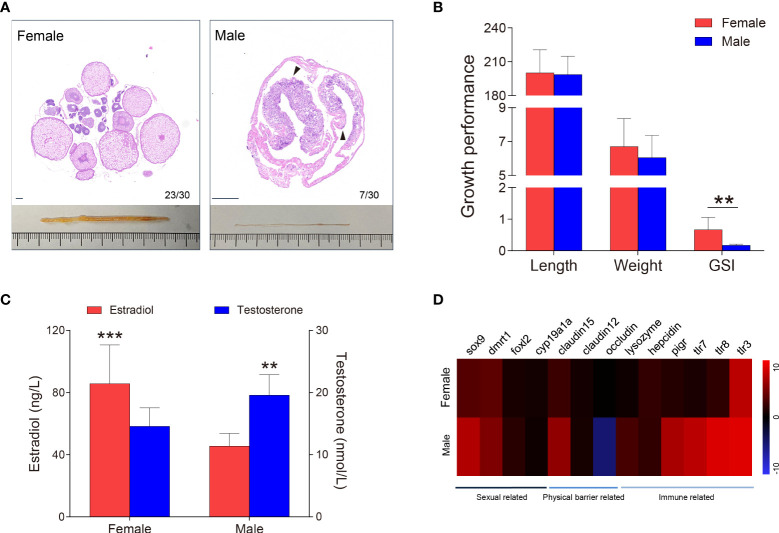
Sex identification of *M. albus*. **(A)** Representative histology graphs (upper) and morphology (lower) from ovary and testis of *M. albus* under the same background. Black triangles indicate genital folds. Scale bar, 100 μm. **(B)** Relationships among body length (mm), weight (g), and GSI (%) of *M. albus*. **(C)** Serum concentrations of estradiol (left) and testosterone (right) in female and male *M. albus.* **(D)** Heat map depicting the relative expression levels of genes in ovary (n=22) and testis (n=5) tissues. **P < 0.01, ***P < 0.001, unpaired Student’s *t*-test. The data are expressed as the mean values ± standard deviation (SD).

Genetic background, nutrition, and environment may affect the microbial community (3). To explore the difference of gonadal bacterial community between female and male in *M. albus*, the sibling F1 generation was generated for eliminating the interference of genetic background **(** **Figure 1A** **)**. Embryos and larval *M. albus* were incubated in the incubator of cell culture room **(** **Figure 1A** **)**, then, the juveniles were feed in the indoor circulating system over fourteen months **(** **Figure 1B** **)**. The influence of nutrition and living environment on individual microbial community is minimized between female and male *M. albus*. To further exclude differences due to individual size, after sex identification, 30 individuals of similar size including females and males were selected for the subsequent study **(** **Figures 1C**, **2A** **)**.

As a natural female-to-male sex reversal freshwater economic fish, the sex of *M. albus* individuals were truly identified. Unexpectedly, the majority of *M. ablus* had female oocytes, but seven individuals had male characteristics, such as the appearance of genital folds and spermatogenic cells **(** **Figures 1A**, **2A** **)**. Given the morphological differences between gonads, we statistically analyzed the growth parameters of the identified *M. albus*. Although no significant differences were observed in body length or weight, a remarkable difference in GSI was detected between female groups and male *M. albus* (**Figure 2B**). Changes in gonadal steroid concentrations typically accompany gonadal transition in species that undergo sex reversal. In the present study, serum estradiol was approximately 2-fold higher in females (85.74 ± 24.30 ng/μL) than in males (45.50 ± 7.59 ng/μL). However, serum testosterone in males reached 19.58 ± 3.05 nmol/L, which was significantly higher than measured in females (14.59 ± 2.88 nmol/L) (**Figure 2C**).

Several genes were then quantified in testis and ovary samples *via* RT-qPCR, including sexual-related genes (cyp19a1a, foxl-2, sox-9, and dmrt-1), physical barrier-related genes (occludin, claudin-12, and claudin-15), and immune-related genes (polymeric immunoglobulin receptor (pIgR), toll-like receptor (TLR-3, TLR-7 and TLR-8), lysozyme and hepcidin) (**Figure 2D**). As expected, the female-related gene foxl-2 was highly expressed in ovaries, whereas the expression levels of male-related genes sox-9 and dmrt-1 are significantly higher in testes than in ovaries. Tight junctions are vital to the structure of the blood-testicular barrier. We found mRNA level of occludin was significantly downregulated whereas claudin-12 and claudin-15 were upregulated in testes, suggesting that structural integrity differed between testis and ovary. Similarly, higher expression levels of TLR-7, TLR-8, and pIgR transcripts were observed in testis than in ovary. Importantly, lysozyme, an antimicrobial peptide, has higher expression in the testes than in ovaries, hinting that the microbial environment may be unique between testes and ovaries.

### 16S rDNA gene sequencing and diversity analysis in *M. albus*

**Figure 3:**
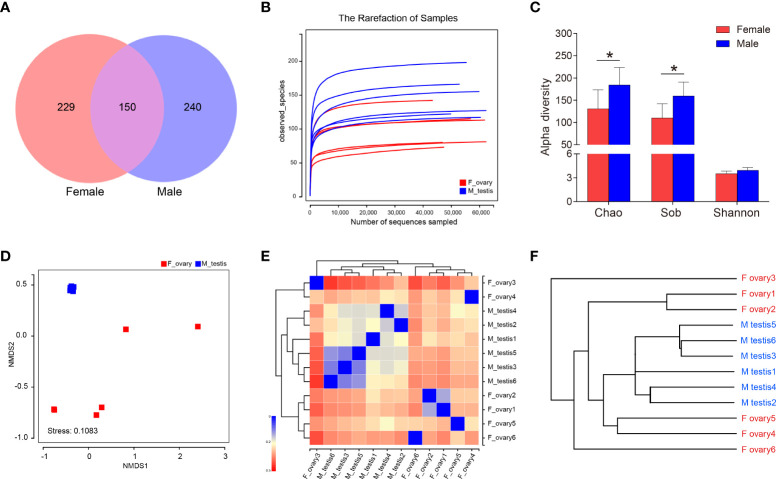
Diversity analysis in *M. albus*. **(A)** Venn diagram displays the number of shared and unique OTUs in the gonads of female and male *M. albus*. **(B)** Rarefaction analysis of female and male gonads. **(C)** Histograms represent alpha diversity analyses in the gonads of male and female gonads *M. albus*, including Chao, Sob, and Shannon indices. **(D)** NMDS shows the relationship between females and males. **(E)** heat map analysis shows the relationship among female and male samples. **(F)** Hierarchical cluster analysis of weighted-unifrac distances generated from ovary and testis was constructed by UPGMA. *P < 0.05, unpaired Student’s *t*-test. The data are expressed as the mean values ± standard deviation (SD).

To distinguish the anatomical relationships between the gonads and the gut in *M. albus*, Micro-CT investigations was performed and presented the gonad abutted the gut and sequentially terminated in the cloaca, respectively **(** **Supplementary Figure 2** **)**. Subsequently, testes and ovaries of *M. albus* were collected for 16S rRNA sequencing to verify whether gonadal microorganisms are related to sex. After quality filtering and normalization to remove adaptors and low-quality or ambiguous bases, a total of 672,573 high-quality sequences were obtained, equivalent to an average of 56,047 reads per sample. Valid sequences were then clustered, resulting in 619 OTUs with a cutoff value of 97% identity. Sequences were classified taxonomically for downstream analysis. Males and females shared 150 OTUs; males had 240 unique OTUs, and females had 229 (**Figure 3A**). A rarefaction curve and coverage indices were applied to evaluate the depth of sequencing and species richness. Our result revealed that most of the OTUs were detected in all samples, and all samples reached saturation (**Figure 3B**). Meanwhile, coverage indices among all samples were as high as 99%, indicating that the sequencing depth was sufficient (**Table 1**). OTUs were then used to analyze differences in the gonadal microbial abundance, and community diversity was compared between female and male fish. Chaos and Sob indices were substantially higher in testis than in ovary samples. Although the difference did not reach significance, the Shannon index was higher in testis than in ovary samples (**Figure 3C**, **Table 1**). These results suggest that sex influences the microbial richness and diversity of gonadal flora in the *M. albus*.

**Table 1:** Alpha diversity of the gonad microbial community in *M. albus*.

	F_ovary	M_testis	*P-*vaule
sobs	110.33 ± 28.88	159.67 ± 28.45	0.0215*
chao	130.89 ± 38.80	184.33 ± 35.54	0.0465*
ace	134.02 ± 29.43	178.72 ± 25.84	0.0288*
shannon	3.51 ± 0.30	3.93 ± 0.33	0.0600
simpson	0.06 ± 0.03	0.04 ± 0.02	0.2631
coverage	0.999 ± 0.0001	0.999 ± 0.00001	0.0663

The data are expressed as the mean values ± standard deviation (SD). P-values were determined using Student’s t-test (*P < 0.05).

Based on the OTU abundance of each sample, non-metric multidimensional scaling analysis ordination was performed to define the relationship between the female and male groups. We found testis samples were similar to each other and could be distinguished from ovary samples, indicating that significant differences in community structure exist between male and female *M. albus* (**Figure 3D**). Hierarchical clustering trees and heat map analysis revealed that samples were clustered into two distinct groups depending on sex **(** **Figures 3E, F** **)**. In addition, PCA and PCoA yielded similar results, suggesting that differences between samples were primarily due to sex **(** **Supplementary Figure 3** **)**.

### Composition of gonadal microflora in female and male *M. albus*

**Figure 4:**
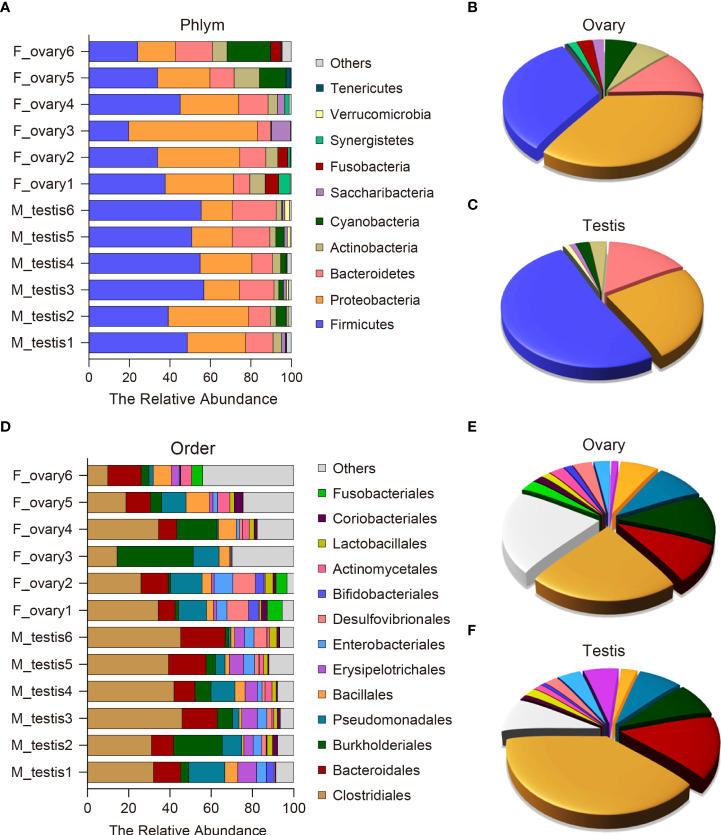
Composition and distribution of bacterial microbiomes in *M. albus* gonads. **(A)** Comparison of the composition and relative abundance of dominant bacterial taxa between ovary and testis at the phylum level. **(B, C)** Pie graphs represent the average relative abundance of each phylum in ovarian **(B)** or testicle **(C)** bacterial community. **(D)** Comparison of the composition and relative abundance of dominant bacterial taxa between ovary and testis at the order level. **(E, F)** Pie graphs represent the average relative abundance of each order in ovarian **(E)** or testicle **(F)** bacterial community.

To further analyze the microbial composition differences between the female and male *M. albus*, microbial sequences from testis and ovary tissues were classified by phylum, class, order, family, and genus. The results showed that eleven phyla were predominantly observed in ovary and testis samples, with Firmicutes, Proteobacteria, Bacteriodetes, and Actinobacteria being the most dominant phyla among females and males. Notably, Proteobacteria, Actinobacteria, and Cyanobacteria were more abundant in the ovarian bacterial community (35.3%, 6.4%, and 5.8%, respectively) compared with the bacterial community in testis (24.5%, 3.2%, and 2.4%, respectively) (**Figures 4A, B**). In turn, a more abundant number of Firmicutes were more abundant in testis (51.0%) than in ovary samples (32.4%). Although the differences between groups did not reach significance, Bacteroidetes was predominant and accounted for 12.0% of the diversity in the ovarian bacterial community and 15.3% in the testis. It is worth noting that Fusobacteria was detected only in female individuals, although its abundance was low (2.8%) (**Figures 4A, C**).

At the order level, potentially pathogenic bacteria were detected in ovary and testis samples, including members of the orders Burkholderiales (11.4% in ovary versus 8.2% in testis) and Pseudomonadales (9.3% in ovary versus 7.9% in testis). In contrast, the orders Erysipelotrichales and Enterobacteriales were significantly enriched in the testis (6.5% and 4.2%, respectively) compared to the ovary (1.3% and 3.1%, respectively) (**Figures 4D-F** and **Supplementary Figure 4**). The testis microbial community was also enriched in potentially beneficial taxa known to produce short-chain fatty acids (SCFAs), including Clostridiales (39.3% in testis versus 23.0% in ovary), Bacteroidales (15.1% in testis versus 9.7% in ovary), and Lactobacillales (2.1% in testis versus 1.5% in ovary). Other SCFA-producing bacteria were enriched in ovaries, such as Bacillales (2.9% in testis versus 7.1% in ovary) (**Figures 4D-F** and **Supplementary Figure 4**). These results indicate that broad, well-defined ranges of bacteria with beneficial and pathogenic characteristics exist in M. albus gonads, and notable differences are related to sex.

### Dominant bacterial community analysis between testis and ovary of *M. albus*

**Figure 5:**
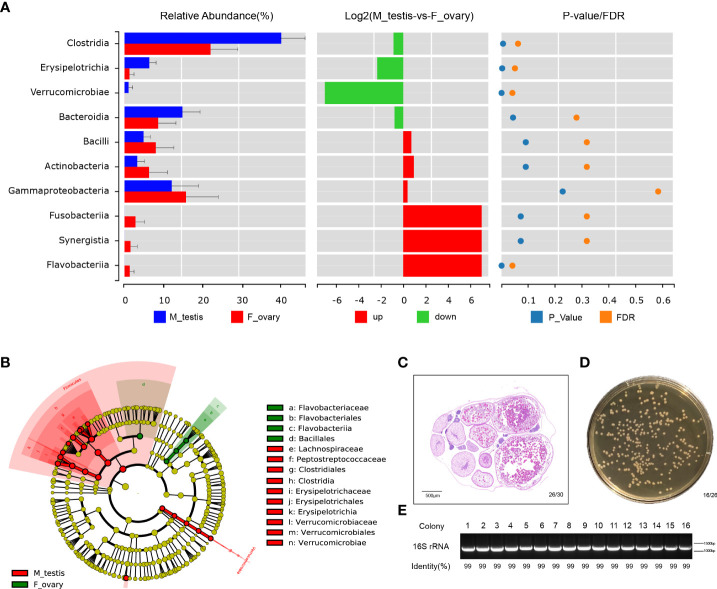
Bacterial community are significantly different between ovary and testis in *M. albus*. **(A)** Differences in microbial communities at the class level. **(B)** Cladogram representation of LEfSe analysis shows that bacterial taxa are significantly associated with the ovary (green) or testis (red). **(C)** Representative graphs of ovary histology in *M. albus*. **(D)** Culture plates from gonads of *M. albus*. Black triangles indicate single colonies of *Bacillus*. **(E)** Agarose gel electrophoresis of colony PCR samples. PCR products were verified by agarose gel electrophoresis and sequencing.

Differences in the microbial communities at the class and genus level were displayed with histograms for the top 10 abundant OTUs from female and male *M. albus*. The relative abundances of *Clostridia* and *Erysipelotrichia* are significantly higher in males than females (*P* < 0.01) at the class level (**Figure 5A** and **Supplementary Figure 5A**). Interestingly, not only are *Clostridium XIVa* and *Bacteroides* significantly different (confirming former studies), but *Escherichia*, a more potentially pathogenic bacterial genus, was identified in testis tissue. Moreover, the beneficial taxa *Romboutsia* were detected significantly in males (**Supplementary Figure 5B**).

Dominant microbiomes that contribute to bacteria differences were also investigated by linear discriminant analysis of effect size (LEfSe). In contrast to male *M. albus*, we observed that the genus *Flavobacteriaceae*, the order *Flavobacteriales*, and the class *Flavobacteriia* were predominant in the female *M. albus*. Meanwhile, the order *Bacillales* (a member of the Firmicutes phylum) was also enriched in the ovary (**Figure 5B**). However, three other members of Firmicutes (*Clostridiales* and *Erysipelotrichales*) were more prevalent in the testis. *Verrucomicrobiales* was other orders that are more predominant in *M. albus* testis (**Figure 5B**). Gonads of *M. albus* propagated under identical conditions were randomly selected for bacterial identification on coating plates to verify the dominant gonadal bacteria. Sex identification revealed ovarian characteristics in almost all gonads, so the tissue homogenates of ovaries were cultured on Brain Heart Infusion Agar, and bacterial colonies with the characteristic white, large and flat morphology were detected on most plates (**Figures 5C, D**). Subsequently, single colonies were isolated and sequenced using 16S rRNA universal primers. The results revealed that the similarity with *Bacillus* was over 99%, which provided additional support that *Bacillus* may be the dominant bacteria in *M. albus* ovaries (**Figure 5E**). These findings indicate that sex exerts profound and complex effects on the microbial community composition and affects dominant bacteria in gonads of *M. albus*.

### Functional prediction of microbial communities in *M. albus*

**Figure 6:**
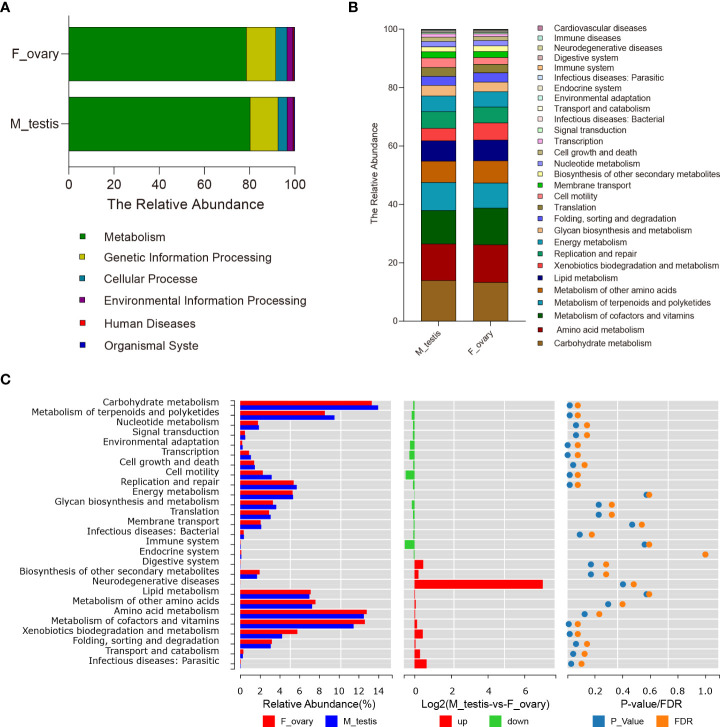
Predictive analysis of microbiota function in gonads of the *M. albus*. **(A)** Average relative abundances in predicted functional genes of gonadal bacterial community at KEGG level 1. **(B)** Average relative abundances in predicted functional genes of gonadal bacterial community at KEGG level 2. **(C)** Average relative abundances and differences in predicted functional genes of gonadal bacterial community at KEGG level 2.

PICRUSt was carried out to predict the functions of genes expressed in the microbial communities in *M. albus* gonads. Using level 1 KEGG ortholog function predictions, three functional categories were significantly different (*P* < 0.05) between females and males: metabolism, cellular processes, and organismal systems (**Figure 6A** and **Supplementary Figure 6**). Concretely, metabolism was more abundant in females, whereas categories involved in cellular processes and organismal systems were more abundant in males. Additional differences between males and females were identified when metabolic pathways were characterized at level 2 KEGG. The functional categories enriched in female bacterial microbiota included the metabolic pathways of xenobiotics biodegradation and metabolism, amino acid metabolism, and metabolism of cofactors and vitamins, whereas the functional categories enriched in males were carbohydrate metabolism and metabolism of terpenoids and polyketides. Interestingly, in males, functional genes in enzyme families, transcription, and cell motility categories were more concentrated. Although the difference between males and females did not reach statistical significance, genes associated with bacterial community involved in immune system function were substantially higher in males, whereas genes involved in immune diseases were enriched in females (**Figures 6B, C**).

## Discussion

Microbiomes in vertebrates vary in a tissue-specific manner and play vital roles in many biological functions, including growth enhancement, nutrition, development, metabolism, and immune responses (31, 32). In mammals, bacterial microbiota could alter gonadal maturation and drive sex-specific differences in gene expression and metabolism (7, 10–12). Although teleost bacterial microbiomes have been extensively studied (5, 20), the diversity present in gonads for most teleost species remains unexplored. In this study, we compared the microbial community compositions of female and male *M. albus*, a teleost that undergoes sex reversal naturally, and predicted the corresponding functional differences.

Environmental and host genetic factors shape individual variations in host-associated microbiota community structures (33). In this study, *M. albus* with identical genetic backgrounds were hatched from embryos and kept under identical conditions (e.g., environment, diet), to minimize any effects caused by environment and heredity. It is generally believed that it takes more than two years for *M. albus* to undergo sex reversal in natural environments (22). Unexpectedly, seven individuals with male characteristics (e.g., genital folds and spermatogenic cells) emerged approximately one-year post-hatching. Previous studies found that temperature affects sex differentiation in various teleost species, so one reason for the early reversal in *M. albus* might be the suitable indoor temperature (34, 35). In our study, significantly higher serum testosterone in males than females provided additional support to the facticity that males had emerged. However, lower average concentrations of estradiol and testosterone were detected in our study, a finding that is at odds with the content of sex hormones reported in a previous study. On the one hand, the environment may be associated with the synthesis, secretion, or transport of hormones (36). On the other hand, differences in the weight and shape of *M. albus* individuals can cause differences in serum hormones according to previous study (37). As assessed in several species, a shared characteristic among fish species in which temperature can alter sex ratios is that exposure to heat during early development upregulates the expression of genes related to testis differentiation with a concomitant down-regulation of genes related to ovarian differentiation (38, 39). We also observed substantially elevated expression of sox-9 and dmrt-1 genes in male testis, further suggesting temperature may have an effect on sexual reversal. Meanwhile, consistent with the previous studies on sex regulated genes, a lower expression of sox-9 and dmrt-1 were also detected in the ovary, which may be caused by the same genome shared between female and male individuals (40, 41). The claudin family of membrane proteins play vital roles in tight junctions structure, an intercellular junction critical for building the epithelial barrier and maintaining epithelial polarity (42). Previous studies in fish have found that upregulation of occludin mRNA levels stabilizes intercellular structural integrity, whereas upregulation of claudin mRNA levels disrupts the structural integrity of cells (43). In this study, we observed that claudin-15 mRNA levels were significantly upregulated and occludin mRNA levels were downregulated in *M. albus* testes, implying that the physical barrier integrity in testis was less than that in ovaries. In vertebrates, TLRs can distinguish among classes of pathogens and serve an important role in orchestrating the appropriate adaptive immune responses. It has been illustrated that TLR3, TLR7, and TLR8 are primarily located in the endoplasmic reticulum and in lysosomal-like vesicles and are thought to have a vital role in anti-viral immunity (44). In the present study, higher expression levels of these TLRs in testis suggest the microbial immune environment may be different between testes and ovaries. Interestingly, pIgR, a gene involved in teleost mucosal defense, exhibited significantly higher expression in testis than in ovary, providing additional support that genes associated with immune responses differ between the sexes, similar to mammals, and these differences likely contribute to sex-specific vaccine outcomes (45). Whether there is a relationship between physical barrier integrity and immune environments, and the specific mechanism needs additional research and development. Lysozyme is an antimicrobial peptide that is widely distributed in teleost and contributes greatly to antibacterial defense due to its ability to cleave the glycosidic bond between N-acetylmuramic acid and N-acetylglucosamine residues in bacterial cell wall peptidoglycans (5, 46). Our data showed that the lysozyme expression increased significantly in testis, indicating that more potential bacterial microorganisms inhabited in testis.

To further investigate the precise differences of gonadal microorganisms, the testis and ovaries of *M. albus* were collected for 16S rRNA sequencing and analyzed, respectively. In contrast to results reported for *M. albus* intestinal flora, this study found that gonadal tissues exhibited higher Alpha diversity in males than females (16). It supported that different tissues are inhabited by uniquely different microbial communities and proportions of specific bacteria (13, 14). Therefore, it was the body site that led to the difference of diversity and richness to a large extent, and it does not rule out that group differences contribute a small amount to the observed diversity. Meanwhile, Beta diversity analysis where the male sample was clustered while the female sample was scattered indicates that the gonadal flora of *M. albus* changes at different developmental stages. The more obvious differences between male and female *M. albus* were reflected in the composition of gonadal microflora (**Figure 4**). Gonad tissues from humans are characterized by Proteobacteria, Firmicutes, Bacteroidetes, and Actinobacteria as the dominating phyla, similar to the microbial compositions observed in this study on *M. albus* (11, 47). However, there are significant differences in the proportion of these microorganisms between testis and ovary. In our study, Firmicutes was the most prominent bacterial community in testis, whereas the relative abundance of Bacteroidetes had no significant difference between the two groups. Although the concept that an increment in the relative abundance of Firmicutes and Bacteroidetes may be associated with obesity has been consistently supported by numerous studies, a recent meta-analysis concluded that there were no statistically significant differences in the Firmicutes/Bacteroidetes ratio between obese and normal-weight adults (48, 49). Combined with our previous results on weight and the recently published meta-analysis, it may further strengthen the deduction that the Firmicutes/Bacteroidetes ratio may not be a robust marker for obesity (50). However, an increased prevalence of the bacterial phylum Proteobacteria is a sensitive marker for an unstable microbial community (dysbiosis) and a potential diagnostic criterion for disease (51). Whether females may suffer from more diseases than males deserves additional study to understand due to the higher proportion of Proteobacteria in females than males observed in the present study. In addition to Proteobacteria, Cyanobacteria also produce a wide variety of potentially toxic secondary metabolites and various other cyanobacterial bioactive compounds that could affect fish health. Previous studies have shown that Cyanobacteria may be related to the effect of environmental stress on metabolic divergences in fish (52). The design of this study eliminated environmental differences; therefore, the relative abundance of Cyanobacteria in ovary relative to testis indicates that there may be intrinsic metabolic differences between *M. albus* males and females.

SCFA synthesized from carbohydrates and indigestible oligosaccharides are rich energy sources for the host metabolism. Intestinal members of the orders *Clostridiales, Bacteroidales*, and *Lactobacillales* are correlated with the biosynthesis and absorption of SCFA by enzymes such as glycosyl transferases, glycoside hydrolases, and polysaccharide lyases (53). Moreover, a compensatory relationship between testicular and intestinal microbiota has been reported (21); therefore, we speculate that gonadal bacterial microbiota are also involved in host metabolism and homeostasis maintenance. Significant differences in the metabolism between the ovary and testis are suggested by the overrepresentation or underrepresentation of the predicted KEGG pathways associated with different metabolic processes and biosynthesis in the ovary and testis. For example, higher levels of microbial functional genes associated with the metabolism of cofactors and vitamins were detected in ovaries, whereas the level of carbohydrate, terpenoids and polyketides metabolism was significantly elevated in testis. Because activated T cells mediate metabolic reprogramming, promote the production of glycolytic flux and lactate, and elevate the production of lipids, proteins, nucleic acids, and other carbohydrates (i.e., induction of biomass), we surmise that these metabolic differences between male and female *M. albus* may be related to their correlative immune function (54). Evidence is accumulating to support specific roles for bacterial community in the development and function of T cells and T regulatory (Treg) cells (53, 55). In the case of distinct *Clostridia* clusters, it could be either independent of pattern recognition receptors (PRRs) or dependent on My-D88 dependent mechanisms (56). In the case of *Bacillus*, induction of Treg cells appears to be mediated by polysaccharide A-induced TLR2 signaling (57). The higher relative abundance of these immune-related bacteria in testis may indicate that males have a more active immune response. Moreover, microbial cell wall peptidoglycans were reported to maintain tight junctions by TLR2-mediated signaling, suggesting that *Bacillus* may be responsible for the integrity of ovary (58). More importantly, certain SCFAs (e.g., butyrate) have been implicated in the development and function of Tregs. Whether there is a certain relationship among microbiota, metabolism, and immunity in male and female *M. albus* needs to be further explored. In addition to *Bacillus*, *Lactobacillus* has also been reported as an aquaculture probiotic, and this study found it to be more abundant in *M. albus* testes. Previous studies have reported that *Lactobacillus* and *Clostridia* are associated with semen quality and fertility status (10, 11). In view of the special characteristic of sexual reversal in *M. albus*, additional studies should focus on the relationship between probiotics and sperm quality in *M. albus*.

This study is the first comprehensive characterization of the microbial communities in *M. albus* gonads to our knowledge. To summarize, this study found significant differences in the microbial composition and distribution of *M. albus* between testis and ovary, which may be relevant to the difference in the metabolism, immune modulation, and host-microbe interactions between female and male groups. These findings provided unique resources for further explore how gonadal bacterial community influences sex-specific differences. Meanwhile, it also provides theoretical support for the improvement of polycystic ovary syndrome.

## Data availability statement

The datasets presented in this study can be found in online repositories. The names of the repository/repositories and accession number(s) can be found in NCBI with the accession number PRJNA832434.

## Ethics statement

The animal study was reviewed and approved by Institute of Hydrobiology, Chinese Academy of Sciences.

## Author contributions

DL conceived the project. DL and KM designed the study. KM performed most of experiment and analyses. XL helped in breeding and sampling. HL and FL helped in analyzed the image data. HC, ZX and YS helped in data analyses. KM and DL prepared the draft and final version of the manuscript. All authors read and approved the final manuscript.

## Acknowledgments

We would like to thank Yuanli Zhao, Rui Li and Meidi Hu for their help in experimental process; Xin Wang, Yuan Xiao and Yan Wang (The Analysis and Testing Center of Institute of Hydrobiology, Chinese Academy of Sciences) for their support of instrument platform and technical. This work was supported by grants from the National Natural Science Foundation of China (Grant No.31922085 to DL), and Natural Science Foundation of Hubei Province (Grant No. 2020CFA056 to DL).

## Conflict of interest

The authors declare that the research was conducted in the absence of any commercial or financial relationships that could be construed as a potential conflict of interest.

## Publisher’s note

All claims expressed in this article are solely those of the authors and do not necessarily represent those of their affiliated organizations, or those of the publisher, the editors and the reviewers. Any product that may be evaluated in this article, or claim that may be made by its manufacturer, is not guaranteed or endorsed by the publisher.
