# Supplementary material for: Gonadal bacterial community composition is associated with sex-specific differences in swamp eels (Monopterus albus)
Source: Front Immunol. 2022 Aug 24;13:938326. doi: 10.3389/fimmu.2022.938326 (PMC9449807; doi:10.3389/fimmu.2022.938326)
Supplement: Supplementary file 1 [file Presentation_1.zip › Supplementary/Supplementary Figure 1.docx]

**
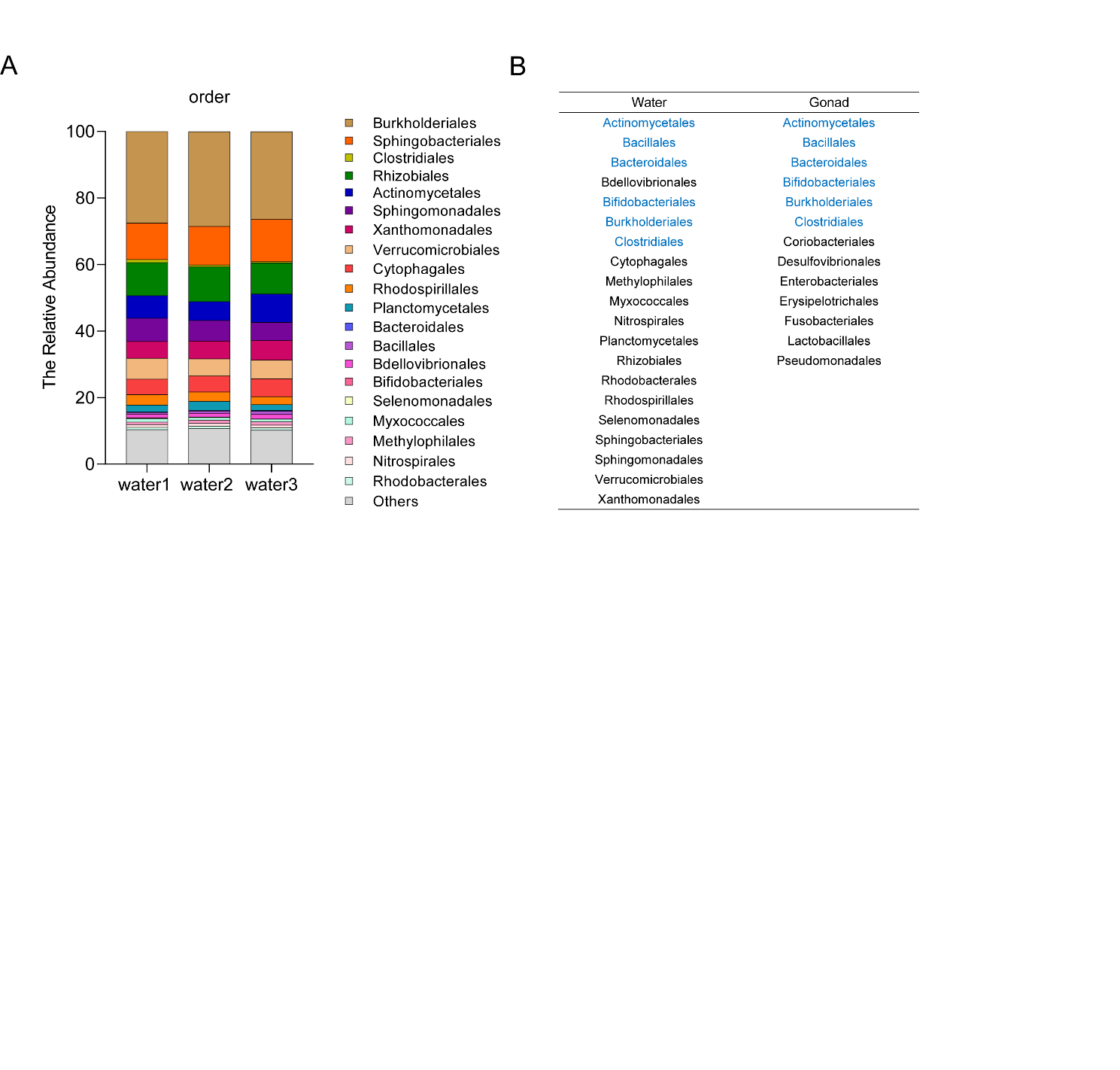
**

**FIGURE S1** | Composition of bacterial microbiomes in water environment of host living **(A)** and comparison of microbial differences in water and gonads **(B)**. Bacteria in blue and black represent the shared and unique microbial community between water and gonads, respectively.
