# Supplementary material for: Gonadal bacterial community composition is associated with sex-specific differences in swamp eels (Monopterus albus)
Source: Front Immunol. 2022 Aug 24;13:938326. doi: 10.3389/fimmu.2022.938326 (PMC9449807; doi:10.3389/fimmu.2022.938326)
Supplement: Supplementary file 1 [file Presentation_1.zip › Supplementary/Supplementary Figure 2.docx]

**
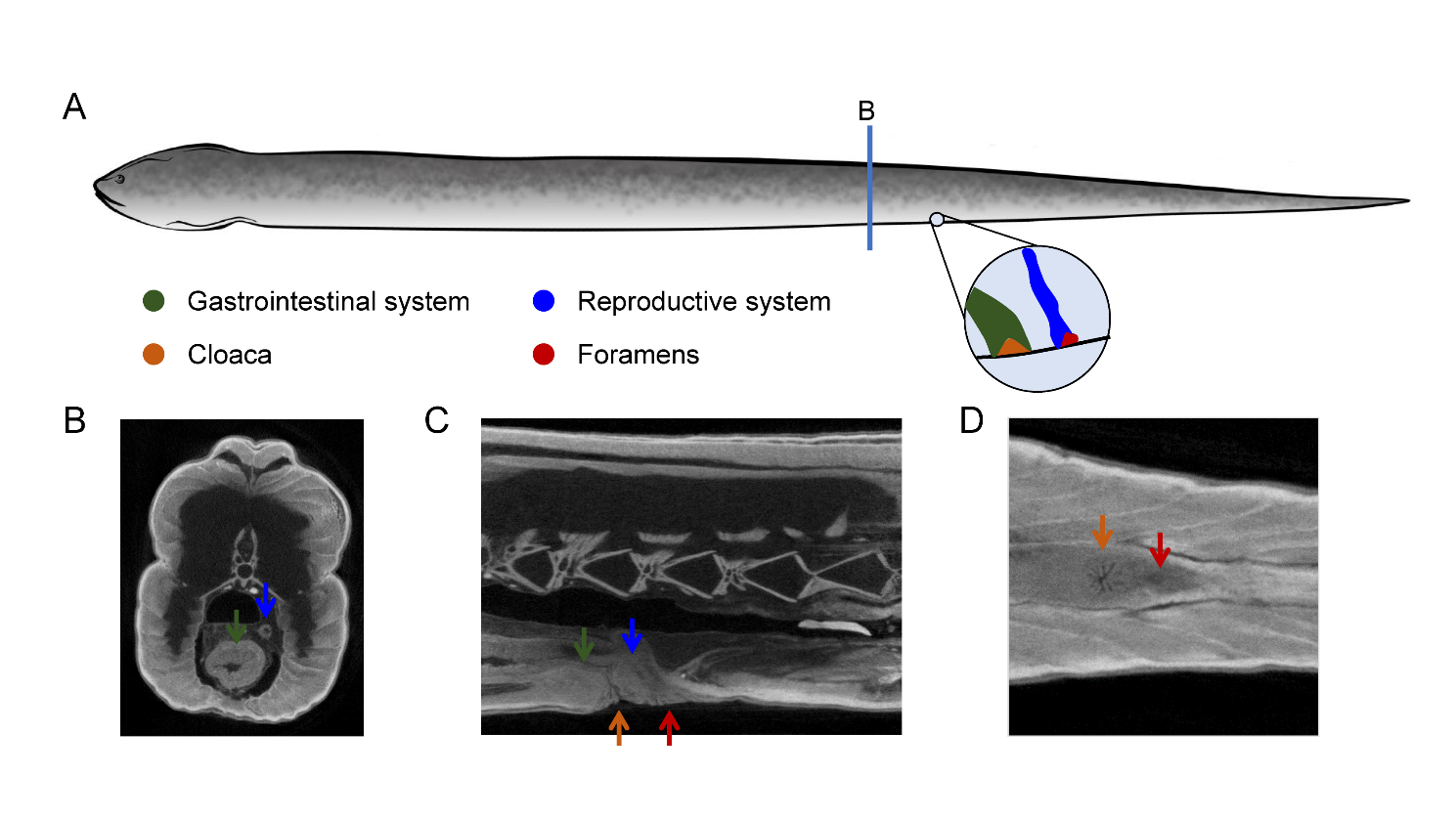
**

**FIGURE S2** | Schematic illustrations the cloacal region. **(A)**. Schematic illustrations the cloacal region of *M. albus*. **(B-D)**. The lateral view (B), front view (C), and vertical view (D) of the cloacal region in *M. albus*, respectively. The green and blue arrows represent the intestine and gonad, respectively.
