# Supplementary material for: Gonadal bacterial community composition is associated with sex-specific differences in swamp eels (Monopterus albus)
Source: Front Immunol. 2022 Aug 24;13:938326. doi: 10.3389/fimmu.2022.938326 (PMC9449807; doi:10.3389/fimmu.2022.938326)
Supplement: Supplementary file 1 [file Presentation_1.zip › Supplementary/Supplementary Figure 5.docx]

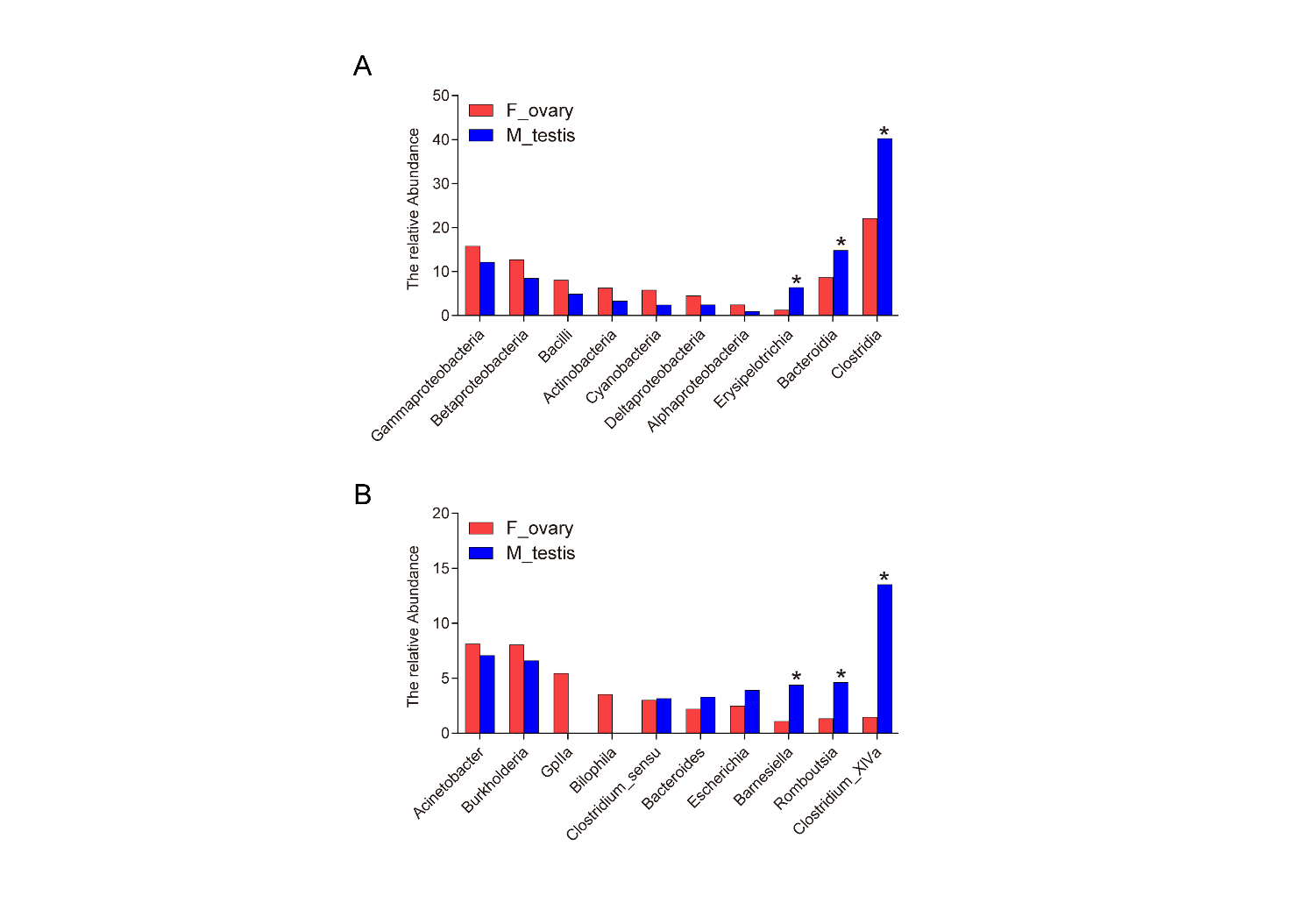


**FIGURE S5** | Comparison of average relative abundances and significant differences among the top 10 species in class level **(A)** and genus level **(B)**. Asterisks indicate significant differences between ovary and testis.
