# Supplementary material for: Gonadal bacterial community composition is associated with sex-specific differences in swamp eels (Monopterus albus)
Source: Front Immunol. 2022 Aug 24;13:938326. doi: 10.3389/fimmu.2022.938326 (PMC9449807; doi:10.3389/fimmu.2022.938326)
Supplement: Supplementary file 1 [file Presentation_1.zip › Supplementary/Supplementary Figure 6.docx]

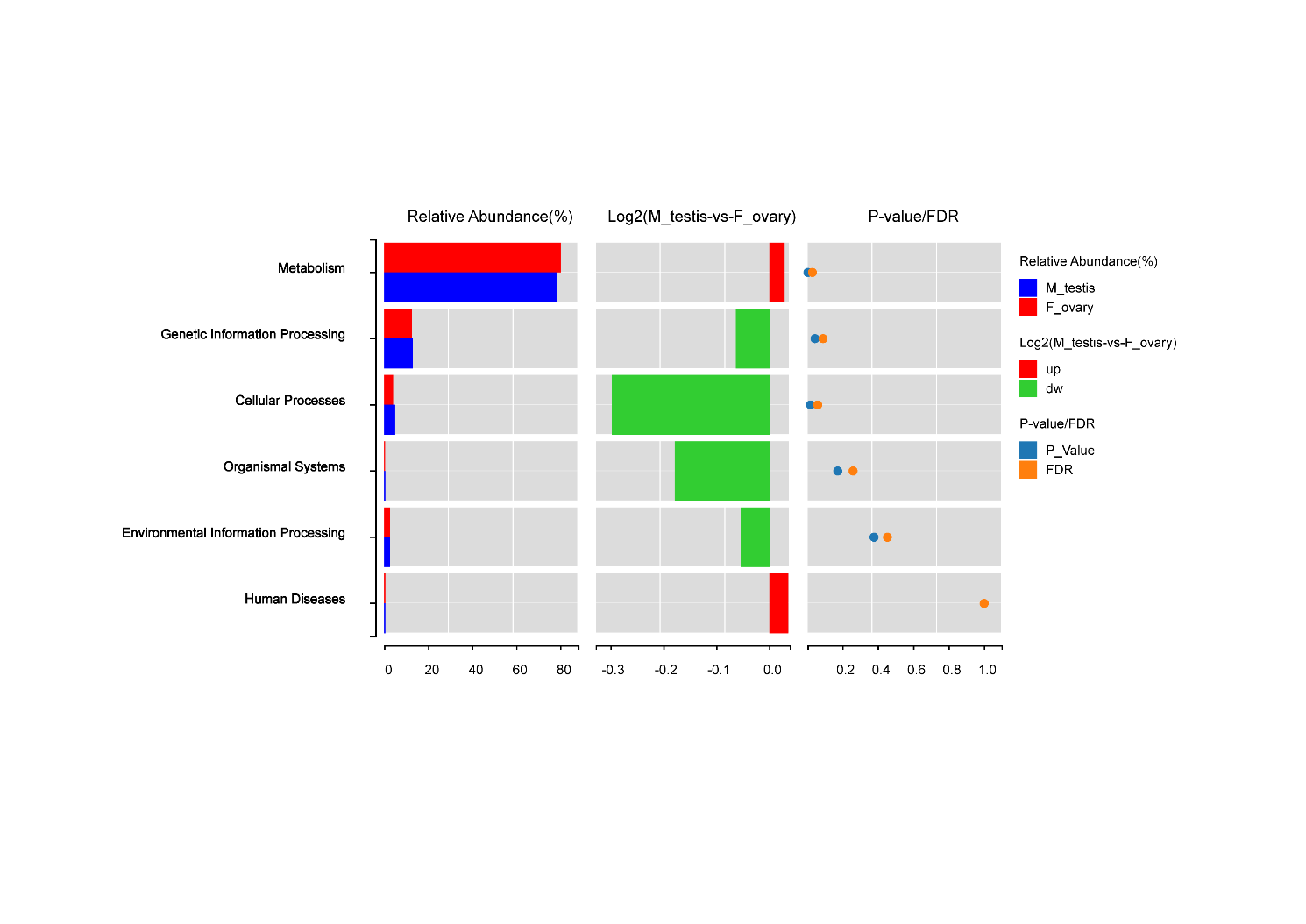


**FIGURE S6** | Average relative abundances and differences in predicted functional genes of gonad microbiota at KEGG level 1.
