# Supplementary material for: Gonadal bacterial community composition is associated with sex-specific differences in swamp eels (Monopterus albus)
Source: Front Immunol. 2022 Aug 24;13:938326. doi: 10.3389/fimmu.2022.938326 (PMC9449807; doi:10.3389/fimmu.2022.938326)
Supplement: Supplementary file 1 [file Presentation_1.zip › Supplementary/Supplementary Table 1.docx]

**TABLE S1 |** Primers used in this study.

| **Gene** | **GenBank**  **accession no.** | **Primer Sequence (5’-3’)** | **Tm**  **(◦C)** | | **Amplicon**  **Length (bp)** |
| --- | --- | --- | --- | --- | --- |
| **REFERENCE GENE** | | | | | |
| EF-1a | KC_011266.1 | F: CGCTGCTGTTTCCTTCGTCC  R: TTGCGTTCAATCTTCCATCCC | | 58 | 102 |
| **SEXUAL RELATED GENES** | | | | | |
| Sox 9 | XM_020622629.1 | F: ATACCCACATCCTGCACAACG  R: TGAAGATCGCATTTGGAGAG | | 58 | 233 |
| Dmrt 1 | XM_020585920.1 | F: CTCGCTGGTTAGCTCTGAT  R: GGAATATGAAACTATCACAAG | | 58 | 189 |
| Foxl 2 | XM_020586693.1 | F: TGACAACAACACGAACAAGGAG  R: GGCAATGAGAGCGACATAGGA | | 58 | 118 |
| Cyp19ala | XM_020605765.1 | F: TACTCAGCAGGTCATCAGCG  R: TCTCATTGACAGGTACACCA | | 58 | 333 |
| **PHYSICAL BARRIER RELATED GENES** | | | | | |
| Claudin 12 | XM_020607277.1 | F: TCACCTTCAATCGCAACG  R: ATGTCTGGCTCAGGCTTATCT | | 58 | 250 |
| Claudin 15 | XM_020611334.1 | F: CTCGCTGCTTGCTTTGACT  R: TTGAAGGCGTACCAGGACA | | 58 | 225 |
| Occludin | XM_020599328.1 | F: TGTCGGGGAGTGGGTAAA  R: TCCAGGCAAATAAAGAGGCT | | 58 | 130 |
| **IMMUNE RELATED GENES** | | | | | |
| PIGR | NW_018127946.1 | F: CAGCATCTTAGCCCAAAC  R: ACCCAGAGTCTTCCACCT | | 58 | 119 |
| TLR 3 | XM_020614353.1 | F: TATTTAGAGCCATACAGGG  R: CACAATCAAGAACGCACA | | 58 | 244 |
| TLR 7 | XM_020596482.1 | F: ATCCTCACGACTTCCCTC  R: TTTCTTTCATCACCCACT | | 58 | 205 |
| TLR 8 | XM_020596483.1 | F: AAGTGAAGCAGGATGAAG  R: AAGTCCCAGATTGAGTGA | | 58 | 139 |
| Lysozyme | XM_020600993.1 | F: GGATGGTTACCGTGGCATCA  R: TAACAACGCGTTTGGCACAG | | 58 | 245 |
| Hepcidin | GU_997139.1 | F: GCCTTTATCTGCATTCTGG  R: CGCAGCCCTTGTAGTTCT | | 58 | 211 |

F: Forward primer; R: Reverse primer.
