# Supplementary material for: Gonadal bacterial community composition is associated with sex-specific differences in swamp eels (Monopterus albus)
Source: Front Immunol. 2022 Aug 24;13:938326. doi: 10.3389/fimmu.2022.938326 (PMC9449807; doi:10.3389/fimmu.2022.938326)
Supplement: Supplementary file 1 [file Presentation_1.zip › Supplementary/Supplementary Table 1.pdf]

TABLE S1 | Primers used in this study.

Gene	GenBank accession no.	Primer Sequence (5'-3')	Tm (◦C)	Amplicon Length (bp)
**REFERENCE GENE**				
EF-1a	KC_011266.1	F: CGCTGCTGTTTCCTTCGTCC<br>R: TTGCGTTCAATCTTCCATCCC	58	102
**SEXUAL RELATED GENES**				
Sox 9	XM_020622629.1	F: ATACCCACATCCTGCACAACG<br>R: TGAAGATCGCATTTGGAGAG	58	233
Dmrt 1	XM_020585920.1	F: CTCGCTGGTTAGCTCTGAT<br>R: GGAATATGAAACTATCACAAG	58	189
Foxl 2	XM_020586693.1	F: TGACAACAACACGAACAAGGAG<br>R: GGCAATGAGAGCGACATAGGA	58	118
Cyp19ala	XM_020605765.1	F: TACTCAGCAGGTCATCAGCG<br>R: TCTCATTGACAGGTACACCA	58	333
**PHYSICAL BARRIER RELATED GENES**				
Claudin 12	XM_020607277.1	F: TCACCTTCAATCGCAACG<br>R: ATGTCTGGCTCAGGCTTATCT	58	250
Claudin 15	XM_020611334.1	F: CTCGCTGCTTGCTTTGACT<br>R: TTGAAGGCGTACCAGGACA	58	225
Occludin	XM_020599328.1	F: TGTCGGGGAGTGGGTAAA<br>R: TCCAGGCAAATAAAGAGGCT	58	130
**IMMUNE RELATED GENES**				
PIGR	NW_018127946.1	F: CAGCATCTTAGCCCAAAC<br>R: ACCCAGAGTCTTCCACCT	58	119
TLR 3	XM_020614353.1	F: TATTTAGAGCCATACAGGG<br>R: CACAATCAAGAACGCACA	58	244
TLR 7	XM_020596482.1	F: ATCCTCACGACTTCCCTC<br>R: TTTCTTTCATCACCCACT	58	205
TLR 8	XM_020596483.1	F: AAGTGAAGCAGGATGAAG<br>R: AAGTCCCAGATTGAGTGA	58	139
Lysozyme	XM_020600993.1	F: GGATGGTTACCGTGGCATCA<br>R: TAACAACGCGTTTGGCACAG	58	245
Hepcidin	GU_997139.1	F: GCCTTTATCTGCATTCTGG<br>R: CGCAGCCCTTGTAGTTCT	58	211

F: Forward primer; R: Reverse primer.
